# Method for conducting systematic literature review and meta-analysis for environmental science research

**DOI:** 10.1016/j.mex.2019.100777

**Published:** 2019-12-19

**Authors:** Wondimagegn Mengist, Teshome Soromessa, Gudina Legese

**Affiliations:** a Department of Natural Resource Management, Debre-Berhan University, Ethiopia; b Center for Environmental Science, Addis Ababa University, Ethiopia

**Keywords:** Method for systematic literature review and meta-analysis studies, Systematic literature review, Meta-analysis, Environmental science, SALSA, PRISMA, PSALSAR, PICOC, Ecosystem services

## Abstract

This paper presents a method to conduct a systematic literature review (SLR) and meta-analysis studies on environmental science. SLR is a process that allowed to collect relevant evidence on the given topic that fits the pre-specified eligibility criteria and to have an answer for the formulated research questions. Meta-analysis needs the use of statistical methods that can be descriptive and/or inferential to summarizing data from several studies on the specific topic of interest. The techniques help to generate knowledge from multiple studies both in qualitative and quantitative ways. The usual method has four basic steps: search (define searching string and types of databases), appraisal (pre-defined literature inclusion and exclusion, and quality assessment criteria), synthesis (extract and categorized the data), and analysis (narrate the result and finally reach into conclusion) (SALSA). However, this work added two steps which are research protocol (define the research scope) and reporting results (stating the procedure followed and communicating the result to the public) at the initial and last step, respectively. As a result, the new method has six basic steps which are abbreviated as PSALSAR. Therefore, this method is applicable to assess the existing knowledge, trends, and gaps in ecosystem services.

In sum, this literature review method presents:

• The PSALSAR method is an explicit, transferable and reproducible procedure to conduct systematic review work.
• It helps to assess both quantitative and qualitative content analysis of the literature review.
• The procedure listed here added two basic steps (protocol and reporting result) on a commonly known SALSA framework.

## Graphical abstract

**Figure:**
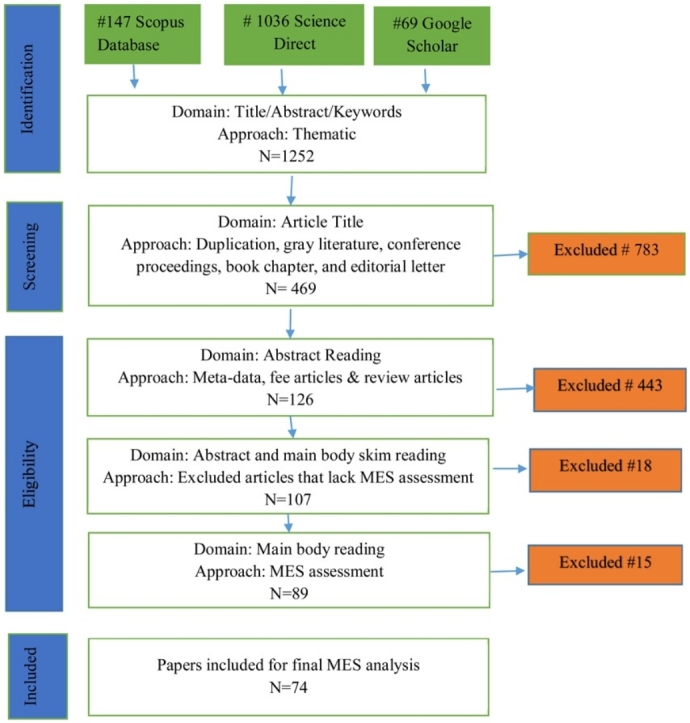

**Specification Table**

**Table:** 

Subject Area	Environmental science, Agricultural and biological science, Natural resource management
More specific subject area:	Environmental science
Method name:	Method for systematic literature review and meta-analysis studies
Name and reference of original method	1). Ecosystem Services Research in Mountainous Regions: A Systematic Literature Review on Current Knowledge and Research Gaps [11]. 2). A systematic review of Augmented Reality content-related techniques for knowledge transfer in maintenance applications [6].
Resource availability	The dataset on ecosystem services research on mountainous regions is publicity available on the web address: https://doi.org/10.1016/j.scitotenv.2019.134581, or https://www.sciencedirect.com/science/article/pii/S0048969719345723

## Method overview

### Rationale

Undertaking a review of the related literature assessment is an important part of any discipline [1]. It helps to maps and assesses the existing knowledge and gaps on specific issues which will further develop the knowledge base. Systematic literature review (SLR) differs from traditional narrative reviews by adopting a replicable, scientific and transparent producers. It helps to collect all related publications and documents that fit our pre-defined inclusion criteria to answer a specific research question. It uses unambiguous and systematic procedures to minimize the occurrence of bias during searching, identification, appraisal, synthesis, analysis, and summary of studies. When the procedure is done properly and has the minimal error, the study can provide reliable findings and reliable conclusion that could help decision-makers and scientific practitioners to act accordingly [[2], [3], [4]]. Well done procedure for the SLR process is essential and it ensures that the work is carefully planned before the actual review work starts. Whereas, meta-analysis needs to apply statistical techniques to derived results based on multiple related studies of data combination. It can help to generate more precise estimates on the topic under study [5]. The main characteristics of SLR and its associated procedure, meta-analysis, are: (i) clearly set the research question which the study would answer, (ii) having a clearly stated objectives that have an explicit and reproducible method; (iii) a searching strings that includes all related studies that would meet the eligibility criteria, and (iv) an assessment of the quality/validity of the selected studies (e.g., assessment of risk of bias and confidence in cumulative estimates), (v) systematic presentation and synthesis of the extracted data from the selected studies, and (vi) making the study findings are available for scientific purpose and decision making [5,6].

This sample SLR work, therefore, aims to enhance our understanding about the existed scientific knowledge and research works on ecosystem service, the ecosystem to sustainably supporting the continuing human well-being, and the main limitations and gaps that hinder the assessment of ecosystem service, and the way forward for future research works. SLR is defined as a “systematic, explicit, and reproducible method for identifying, evaluating, and synthesizing the existing body of completed and recorded work made by researchers, scholars, and practitioners” [6]. According to Grant and Booth [7], the framework of Search, Appraisal, Synthesis, and Analysis (SALSA) is a methodology to determine the search protocols which the SLR should follow. This guarantees methodological accuracy, systematization, exhaustiveness, and reproducibility. Most scientific work [[6], [7], [8], [9]] applied this methodological approach to reduce risks related to publication bias and to increase its acceptability of the work. Thus, most review works followed the literature search protocol of Preferred Reporting Items for Systematic Reviews and Meta-Analyses [10] and the framework of Search, Appraisal, Synthesis, and Analysis (SALSA) [7]. From those common review method types, this article authors associated *Protocol* and *Reporting result* with Search, Appraisal, Synthesis, and Analysis framework, and develop (PSALSAR) framework. This PSALSAR framework of SLR work, therefore, applied six steps and their description is presented in Table 1. Each SLR method steps and their outcomes are explained in detail in the following subsections using the SLR done by Mengist et al. [11] on ecosystem services research in mountainous regions as a case study.

**Table 1:** The frameworks for systematic and meta-analysis studies.

	Steps	Outcomes	Methods
PSALSAR Framework	Protocol	Defined study scope	Only the mountain ecosystem and its various ecosystem services
	Search	Define the search strategy	Searching strings
		Search studies	Search databases
	Appraisal	Selecting studies	Defining inclusion and exclusion criteria
		Quality assessment of studies	Quality criteria
	Synthesis	Extract data	Extraction template
		Categorize the data	Categorize the data on the iterative definition and ready it for further analysis work
	Analysis	Data analysis	Quantitative categories, description, and narrative analysis of the organized data
		Result and discussion	Based on the analysis, show the trends, identify gap and result comparison
		Conclusion	Deriving conclusion and recommendation
	Report	Report writing	PRISMA methodology
		Journal article production	Summarizing the report result for the larger public

### Method details: the six basic steps

#### Protocol – SLR methodology step 1

The need for a research protocol for SLR is for the consideration of transparency, transferability, and replicability of the work, which are the characteristics that make a literature review systematic [12]. This helps to minimize the bias by conducting exhaustive literature searches. Under this stage, the most challenging issue is determining the research scope. Once the research scope is determined, it helps to formulate research questions, and research boundaries to identify the proper research method [6].

The framework of Population, Intervention, Comparison, Outcome, and Context (PICOC) is applicable to determine the research scope. According to Booth et al. [12] description, the PICOC framework together with the definition of each concept is listed in Table 2 and it applies to each SLR step.

**Table 2:** SLR research scope based on the application of the PICOC framework to the determined objectives.

Concept	Definition according to Booth et al. [12]	SLR application
Population	The research work dealing with ecosystem services in mountainous regions.	Scientific research work on ecosystem services from mountainous regions. Mainly on ecosystem services such as regulating, supporting, cultural and provisioning services, as well as ecosystem services trade-offs/synergies.
Intervention	Existing techniques utilized to address the problem identified.	Indicating the gaps that need further research work: for instance, developing an appropriate methodology for ecosystem services that lack methods, integrate ecosystem service studies with human well-being, to study trade-offs between multiple ecosystem services, cover the unstudied mountain regions, less studied MES indicators like pest regulation, pollination, disease regulation.
Comparison	Techniques to contrast the intervention used to measure the ecosystem services against each other.	Difference between the different methods applied to quantify/value/map various MES.
Outcome(s)	Measure to assess the knowledge and gaps mentioned in the selected publications in MES studies.	Existing knowledge on MES such as the most/least studied MES, categories of MES, the methods and model approach used, data types, purpose and the scale of the studies. Mentioned gaps: limitation related to methodological, modeling, data quality, and lack of studies on trade-offs/synergies.
Context	The particular settings or areas of the population.	Trends of MES research, existing knowledge in MES studies, the challenges and gaps in MES, the geographical distribution of existed studies, Study distribution based on categories of MES assessed.

The refined objectives of this SLR on mountain ecosystem services (MES), as a case study are presented in the form of research questions as listed below.

Therefore, the refined research questions were:

1 What is the state-of-the-art in MES?
2 Which MES types had the highest and the least number of studies?
3 Which modeling approaches are common to assess MES?
4 What are the diverse development trajectories and gaps in the sustainability of MES?
5 What are the current challenges impairing MES studies?
6 What are the lesson learned and the way forward for mountain ecosystem studies?

These were the research questions that the study would answer by following the PSALSAR approach [11].

#### Search – SLR methodology step 2

This phase consisted of searching strategy and delivery. The search strategy helps to define appropriate search string and identify the relevant databases to collect the relevant documentation [6]. The number of databases for SLR searches could be defined and restricted, though the number of databases is significantly determined by the nature of the topic area [13]. Therefore, the search string definition should be based on the terminology identified for the population in the SLR application in the PICOC framework (Table 2). The search string was listed in Table 3 and concentrates mainly on the "mountain ecosystem" and "mountain ecosystem services". The following syntax was used: TITLE-ABS-KEY as additional search engine in combinations of the above keywords like "ecosystem services trade-offs", OR "ecosystem services synergies", OR "ecosystem services initiatives", OR "ecosystem services gaps" OR” ecosystem services challenges" OR “ecosystem services modeling” OR “ecosystem services approaches”. Search terms had run in separate or with limited combinations that considered the requirements, or limitations, of the database used. From such databases when publications were not downloaded for further systematic investigations, they were rejected.

**Table 3:** The searching terms used and the total number of publications from each database.

Databases	Searching string and searching terms		No of articles	Date of acquisition
Scopus	Main searching terms-using doc title, abstract, and keywords	“Mountain ecosystem” AND “services”	70	29/6/2019
		“Mountain ecosystem services”	6	15/6/2019
	Secondary searching terms	“Mountain ecosystem” AND “trade-offs”	3	15/6/2019
		“Mountain ecosystem” AND “synergies”	3	15/6/2019
		“Mountain ecosystem” AND “initiatives”	11	15/6/2019
		“Mountain ecosystem” AND “gaps”	17	15/6/2019
		“Mountain ecosystem” AND “challenges”	38	15/6/2019
Science Direct	Main searching terms	“Mountain ecosystem” AND “services”	421	28/6/2019
		“Mountain ecosystem services”	12	15/6/2019
	Secondary searching terms	“Mountain ecosystem” AND “trade-offs”	32	15/6/2019
		“Mountain ecosystem” AND “synergies”	53	15/6/2019
		“Mountain ecosystem” AND “initiatives”	169	15/6/2019
		“Mountain ecosystem services” AND “gaps”	3	15/6/2019
		“Mountain ecosystem” AND “challenges”	346	15/6/2019
Google Scholar	Main searching terms- where all is found in the title of the article	“Mountain ecosystem” AND “services”	34	28/6/2019
		“Mountain ecosystem services”	31	14/6/2019
	Secondary searching terms	“Mountain ecosystem” AND “trade-offs”	1	14/6/2019
		“Mountain ecosystem” AND “synergies”	1	14/6/2019
		“Mountain ecosystem” AND “initiatives”	1	14/6/2019
		“Mountain ecosystem” AND “gaps”	0	14/6/2019
		“Mountain ecosystem” AND “challenges”	1	14/6/2019

N.B. The data here includes reviewed and original articles of all languages.

The search databases for this study were Scopus, science direct and google scholar. The articles were peer-reviewed journals from the three data sources and literature searches were finalized on 29 June 2019. The search was conducted in these various internationally recognized databases to collect relevant information from publications. Science Direct is an online collection of published scientific research operated by the publisher Elsevier, and it is an online academic citation index, at the same time [14]. Scopus is an international database of peer-reviewed publications from all over the world [9]. Google scholar, unlike web of knowledge, science direct and Scopus, does not give a publisher list, journal list, journal types, or any information about the time-span or the refereed status of records. However, using an advanced search engine in Google scholar, it is helpful to cover citations that are not covered by other databases [15]. Based on the search string, each database should be searched and the number of available publications and their acquisition date should be mentioned.

The search delivery step includes the use of the search string to access the selected databases in order to collect multiple related literature papers [6]. By applying of the search string in the selected databases, the number of available literature would be known as search results which were indicated in Table 3. However, the number of articles included in the final analysis was influenced by the searching criteria that the researcher would use and the objective planned to be achieved [16]. Besides, the size and types of databases used for searching related publications can determine the sample size used for analysis [17]. Before conducting the actual systematic review search, a pilot literature search should be done to refine the searching keywords to cover the targeted study objectives [18]. Therefore, a pilot search should be done before determining the actual search engine to refine the searching terms. For instance, during our pilot searching, the result from the selected three databases was large that infers the existence of voluminous of related articles. These were due to the extended time, the application of broader searching strings, and the result from each database was also independent.

Therefore, the article search was restricted to those which were published between the years 1992 and 2019. The reason was that in 1992, the Rio Earth Summit was held and that was the milestone where the international significance of mountains was codified in Agenda 21 Chapter 13 [19]. That was the period when the concept and the term ecosystem and ecosystem service were common. Thus, the application of strict inclusion and exclusion criteria was needed to narrow down the results to the most relevant papers to achieve the objectives of the review work [11].

#### Appraisal – SLR methodology step 3

The appraisal is the phase where the selected articles were evaluated based on the review work objective. The study selection implied screening of the selected literature to identify relevant papers for the review work. It has two basic steps: selecting studies using inclusion criteria and quality assessment.

(I) Selection of related studies

By applying the inclusion and exclusion criteria, papers that fulfill the inclusion criteria were selected for further investigation and content assessments. The predefined literature inclusion and exclusion criteria to achieve this systematic review work were presented in Table 4. Mainly, papers like gray literature, extended abstracts, presentations, keynotes, review articles, and non-English language papers were omitted. There might be publications/articles that cover ES even if they did not mention “mountain ecosystem services/mountain services” in their title, keywords or abstracts. However, these types of articles were not included during the review as they were far from the scope of the review work, which is defining the status quo of the MES.

**Table 4:** SLR study selection of literature using inclusion and exclusion criteria.

Criteria	Decision
When the predefined keywords exist as a whole or at least in title, keywords or abstract section of the paper.	Inclusion
The paper published in a scientific peer-reviewed journal	Inclusion
The paper should be written in the English language	Inclusion
Studies that present pieces of evidence on synergic/tradeoff studies	Inclusion
When the articles address at least one MES indicator	Inclusion
Papers that are duplicated within the search documents	Exclusion
Papers that are not accessible, review papers and meta-data	Exclusion
Papers that are not primary/original research	Exclusion
Papers that got published before 1992	Exclusion

**Fig. 1:**
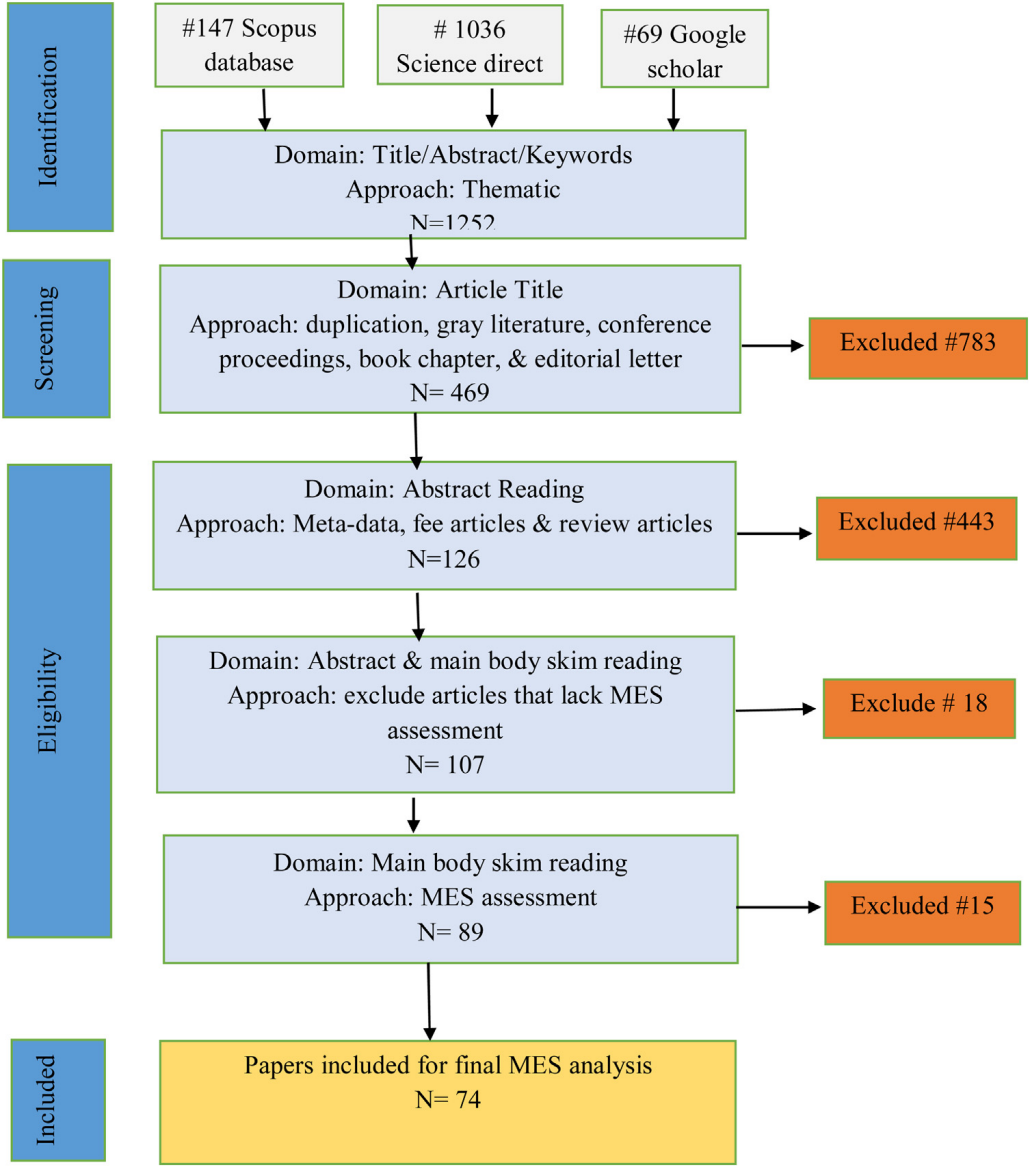
The flow diagram for the database search of publications for systematic reviews.

Source: Modified from Moher et al. [10] and Mengist et al. [11]

The general screening processes and the flow of selecting relevant literature were presented in Fig. 1. In the initial stage, a total of 1252 records were found (69 from Google scholar i.e. using advanced search technique, 1036 from science direct, and 147 from Scopus). After removing of works of literature such as gray literature, extended abstracts, presentations, keynotes, book chapters, non-English language papers, and inaccessible publications, the number of literature was reduced to 469 articles retained for further title reading. After that, only 126 articles fulfilled the eligibility criteria for further abstract reading. After reading the article abstract, only 107 articles were remained for the main body reading. Among them, 89 of them assessed MES and such articles were downloaded for further screening steps. During main body reading, duplicated papers and articles that lack clear ecosystem service assessment methods were manually removed. In the end, 74 publications have remained that fulfilled all the inclusion criteria used in this SLR work (see Fig. 1).

The final list of related publications was downloaded for further analysis. The articles used for further investigation has consisted of 5.9 % of the original articles in the databases. Thus, this review work used a larger sample size compared to Perevochtchikova et al. [9] and Yang et al. [20] that used 0.7 % and 2.8 % of the original number of articles in the databases, respectively. However, the searching criteria and the objectives of the review to achieve determine the sample size of the articles included in the analysis [16]. In addition, the number and types of databases used for searching publication determine the sample size used for analysis. Moreover, most research articles on the topics were not open sources and that limited the final number of published articles included for further assessments.

(I) Quality assessment

Each SLR was evaluated using the following criteria which are based on four quality assessment (QA) questions:

QA1. Are the review’s inclusion and exclusion criteria described and appropriate?

QA2. Is the literature search likely to have covered all relevant studies on the topic?

QA3. Did the selected publication had blind reviewers that assess the quality/validity of the study?

QA4. Was the type of MES mentioned in the publication described adequately?

#### Synthesis – SLR methodology step 4

The synthesis step consisted of both extraction and classification of relevant data from selected papers to derived knowledge and conclusions. The data extraction process involved the identification and extraction of relevant data from the selected papers. Similar to survey research, there is coding for SLR and meta-analysis studies. From the selected published articles, using the prepared criteria, information is extracted by the coder [21].

To address the SLR objectives, the variable of interests was organized on the general characteristics of the articles and on the specific parameters used to evaluate/quantify/map the MES. The general information of the articles include years of publication, analysis types (quantitative, qualitative, mapping or mixed), study types and scale, numbers of ecosystem services assessed, and country or region where the study was conducted. Thus, the case study used ten variables of interest which were defined and indicated in Table 5. Finally, the data related to each selected paper was extracted into an Excel spreadsheet for data processing. The categorization step included the classification and processing of the data extracted to prepare it for further analysis, in which the final result was presented using charts and various types of graphs [11].

**Table 5:** The criteria used for the extraction of information from the selected articles.

No	Criteria	Categories considered	Justification
1	Year of publication	Between 1992–June 2019	Those studies before 1992 were discarded
2	Name of journal	–	To describe the distribution of the work
2	Study site	Name of the country	Geographic site
3	Types of data sources	Primary data	Data derived from sampling in the field (e.g., field data, surveys, or interviews or census data)
		Secondary data	Data types which were derived from other readily available information and not verified in the field (e.g., remote-sensed data, socioeconomic data, and mixed sources like databases like global statistics)
		Mixed data	Database (global statistics, e.g., map of carbon storage and FAO reports), bibliography, modeling, surveys, and field data.
4	Method	Look-up tables	Use of existing MES values from the literature
		Expert knowledge	Experts are invited to rank MES types based on their potential to provide specific ecosystem services to human beings
		Causal relationships	Incorporate existing knowledge to link with related ecosystem processes and the services to create a new proxy layer of the MES
		Models	Employing field data of MES as response variables and proxies (e.g., biophysical data and information obtained from GIS) as explanatory variables.
5	The scale of the study site using Martínez-Harms and Balvanera [22]	Patch	10–10^2^ km^2^
		Local	10^2^–10^3^ km^2^
		Regional	10^3^–10^5^ km^2^
		National	10^5^–10^6^ km^2^
		Global	*>*10^6^ km^2^
6	Mode of assessment	Qualification	Expressing the ecosystem service value with verbal terms
		Quantification	Expressing the ecosystem service values using tons/year/or /hectare
		Economic valuation	publications analyzed monetary value of MES
		Trade-offs	Expressing the changes in different ecosystem services as well as the change in the same ecosystem services between the present and future time
		Mapping and modeling	Studies showing the spatial distribution of the MES
		Combined	The publication used more than one of the above Assessments
7	Types of MES according to Millennium Ecosystem Assessment [23]	Cultural ecosystem services	Both tangible and intangible benefits derived from the ecosystem, such as recreation, aesthetics, spiritual benefits, and so on
		Provisioning ecosystem services	Products obtained from ecosystems, such as water, food, fiber, etc.
		Regulating ecosystem services	Ecosystem services that regulate the environmental conditions in which human beings live (e.g., climate regulation, hydrological cycles, water quality)
		Supporting ecosystem services	Basic ecosystem services that maintain the generation of all other ecosystem services (e.g., soil formation, pollination, nutrient cycling)
8	Number of MES assessed	In number	At least one MES type should be studied: climate regulation, erosion control, water purification, air quality, pest regulation, etc.
9	Purpose of publication	Expansion of site-specific knowledge	Studies describing the MES of the site using monetary and/or biophysical terms
		Methodological development	To develop a new method or to check existed methods on MES
		Management option	To recommend management option to suitable utilization of the resources
		Policy implementation	the publication used existing policies to frame MES as well as discussed possible future policy issues related to MES
10	Difficulties mentioned	Methodological	Uncertainties on the result due to the application of the unclear or less developed method
		Others	Uncertainties linked with lack of conceptual clarity
		Data	Primary and secondary data source quality and scarcity that challenges the work and soon
		Lack of model validation	Most MES studies lack to verify the results using model validation

#### Analysis – SLR methodology step 5

The analysis phase encompassed the evaluation of synthesized data and the extraction of meaningful information and concluding the selected papers. At this phase, the formulated research questions would have answers. It covers both the qualitative and quantitative explanation and narration of the results, making discussion, indicating the way forward about the future research works and inferring a conclusion. The data from the final list of selected articles can be summarized in descriptive and/or basic inferential statistical techniques. The type and use of statistical tools depend on “the nature of the research findings, the type of statistics reported for each study, and the hypotheses tested by the meta-analysis” [21].

In this case study, descriptive statistics were used to calculate the trends of publications, assessing indicator element of MES, date of publication, spatial scale, type of assessments used, the ecosystem that had more and less study coverage. An overview of the evidence, knowledge gaps, and implication for resource conservation were given based on the selected ten criteria used in this study. The systematic review also captures the implication of the state of the research for policy implication and implementation, and the kinds of scientific research needed in the future from various disciplines that has interest and capability to conduct research. The selected studies were classified based on the year of publication, the country where the study conducted, and the spatial scale of the study which is a patch, local, regional, national and global. The other extracted information were the types of MES studied, the purpose of the study, synergistic/trade-off interaction between MES, the limitation mentioned by the paper, and the management option forwarded.

However, according to Shelby and Vaske [21] analysis and result reporting depends on personal judgements of the analyst, researchers understanding level of the research and study purpose. Depending on the nature of the studies, application of statistical analysis tools in SRL and meta-analysis are important and necessary to convey results in reasonable way and also to convince final output users.

The study applied Voyant tool (https://voyant-tools.org/), an open-source, web-based application for performing text mining and analysis which supports scholarly reading and interpretation of texts from the selected articles. It could also employ to assess the frequencies of keywords from all selected published articles.

#### Report – SLR methodology step 6

The report phase of SLR included the description as well as the presentation of the methods followed and results obtained from the selected literature. According to del Amo et al. [6], report phase has two steps: (i) description of the main procedure followed, i.e. explained in Table 4 (ii) public presentation of the result like a journal article. In SLR, a journal article production was the last step and helps to provide the research output for scientific purposes.

## Conclusion

This article showed the basic steps that need to be followed to conduct SLR and meta-analysis studies on environmental science, agricultural and biological science and even for other social science fields. The method can help to generate topic-specific existing knowledge, trends, gaps observed and derived a conclusion that would be appropriate for policymakers and scientific community. Besides, it is easy to replicate the PSALSAR analysis method and can be used by anyone who has an interest to conduct a systematic literature review and meta-analysis work.

## Funding source

No source was secured.

## Declaration of Competing Interest

The authors of this article declare that they have no conflict of interests.
